# The education of traditional Japanese (Kampo) medicine: surveys of training hospitals and residents

**DOI:** 10.1186/s12906-017-1634-2

**Published:** 2017-03-02

**Authors:** Makoto Arai, Yoshinobu Nakada, Shun-ichiro Izumi

**Affiliations:** 1 Department of Kampo Medicine, Tokai University School of Medicine, 143 Shimokasuya, Isehara, Kanagawa 259-1193 Japan; 2 Division of Postgraduate Education, Tokai University Hospital, 143 Shimokasuya, Isehara, Kanagawa, 259-1193 Japan; 3 Department of Obstetrics and Gynecology, Tokai University School of Medicine, 143 Shimokasuya, Isehara, Kanagawa, 259-1193 Japan

**Keywords:** Kampo education, Questionnaire, Survey, Training hospitals, Residents

## Abstract

**Background:**

Japanese physicians prescribe Kampo medicine, but Kampo education is not standardized. We surveyed training hospitals and residents to identify problems and suggest solutions to promote Kampo education during and after residency.

**Methods:**

This was a double questionnaire survey of 1011 training hospitals in Japan and 93 Tokai University School of Medicine graduates of 2011.

**Results:**

There were 816 effective responses (81%) from the training hospitals. Most instructors (84%) thought physicians should have Kampo clinical skills; 67% thought positively about introducing Kampo education into clinical training; 23% of the hospitals provided Kampo education; 70% of instructors at hospitals without Kampo education indicated the lack of Kampo instructors, 16% lack of time, and 7% no necessity for Kampo education; hospitals permitted Kampo education through voluntary study (42%), lectures sponsored by Kampo manufacturers (35%), and study sessions with other hospitals (32%); independent study sessions (10%); smaller hospitals were less active in Kampo education than larger ones. The survey of residents had 72 effective responses (77%): 91% were interested in Kampo medicine; 96% thought it worth learning; 31% could learn it during residency; 52% were not satisfied with the training, 83% wanted to learn it; 73% thought it should be introduced into the curricula; 93% prescribed Kampo medicine, and residents who learned it prescribed it more; 48% were reluctant to prescribe it after residency; 89% thought Western and Kampo medicine should be integrated.

**Conclusions:**

Instructors knew Kampo education was needed, but little of it was taught, especially in small hospitals, because of the lack of Kampo instructors. Residents recognized the need for Kampo medicine and were motivated to learn it. Kampo medicine was mostly prescribed because instructors suggested it. Because of the limited opportunities to learn Kampo medicine, it should be taught during residency. In small hospitals, cooperation with other hospitals could be a solution to teach Kampo medicine.

**Electronic supplementary material:**

The online version of this article (doi:10.1186/s12906-017-1634-2) contains supplementary material, which is available to authorized users.

## Background

Kampo medicine, i.e., traditional Japanese medicine, was introduced into Japan from China via the Korean Peninsula 1500 years ago, and developed into a unique Japanese medicine over a relatively long period of time in the crucible of the Japanese climate and culture [1]. Kampo medicine has, therefore, developed into a system that is completely different from that of traditional Chinese and Korean medicine. Nowadays, complementary and alternative medicine (CAM) attracts attention globally, and Kampo medicine is most frequently practiced and trusted by doctors in Japan among CAM therapies [2, 3]. But in the medical education system in Japan, unlike both China and South Korea, there are no traditional medical schools or a standardized medical licensing system for Kampo medicine. While all Japanese physicians have to learn Western medicine in medical schools [4, 5], under the national health insurance system, they are permitted to prescribe not only Western pharmaceutical medicine but also Kampo medicine. Consequently, in recent years, most Japanese physicians have come to realize that Kampo therapy is clinically useful, prescribe Kampo medicine in the clinical setting [6] and use it in clinical practice [7–9]. In spite of this, learning Kampo medicine during residency is left up to the discretion of the individual residents themselves and the individual educational policy of each training hospital. The main reason for that is because a standardized Kampo educational system has not yet been established in Japan [10]. Most physicians who have not had formal Kampo education learn Kampo medicine by self-study [6], and tend to use Kampo formulae based not on traditional Kampo theory but on western biomedical diagnoses and theory [11]. Their own lack of knowledge, and personal experience with Kampo medicine, may limit their ability to assist many patients who use Kampo medicines [12]. To solve these essential issues, a consistent, standardized Kampo educational system from undergraduate years through 2 years of residency should be promptly established.

We, therefore, initially investigated the current status of Kampo education for undergraduates and residents. Regarding the undergraduate Kampo education, we surveyed the Kampo curricula at all 80 medical schools in Japan and reported the current status and problems to be solved [10]. We also initially conducted a pilot study on the Kampo education in the 58 clinical training hospitals in Kanagawa prefecture, which is adjacent to Tokyo to the southwest [13]. However, a nationwide survey is warranted because the impetus to teach Kampo medicine to residents would most likely vary widely among all the training hospitals throughout Japan. Furthermore, it will be necessary to not only survey the training hospitals where the teaching is done but also to survey the residents who are being taught to more accurately and thoroughly understand the current situation of Kampo education during 2 years of residency. We conducted a questionnaire survey about the changes in awareness of Kampo medicine among the fourth-year medical students of Tokai University School of Medicine before and after their Kampo lectures [14]. However, to date, no follow-up studies about the contents of their Kampo education or any subsequent changes in their awareness of Kampo medicine during their residency have been reported.

The aim of the present study was, therefore, to survey the current status of Kampo education from the two main viewpoints of clinical training: the hospitals where the teaching is done, and the residents who are being taught, in order to identify the major problems and suggest solutions to promote Kampo education during the 2-year residency and beyond.

## Methods

We conducted two surveys on Kampo education during 2 years of residency by postal questionnaires. One was a survey of the clinical training hospitals conducted in Japan from September of 2013 through May of 2015, and the other was a survey of the awareness of Kampo medicine of the residents who graduated from Tokai University School of Medicine in 2011, which was conducted from May 2013 through December of 2014.

### The survey of the clinical training hospitals in Japan

The questionnaire, for all of the 1011 clinical training hospitals in Japan was mailed to the director of each hospital and the instructors responsible for clinical training. The questionnaire asked about the need for Kampo treatment and education, implementation status of Kampo education, and any future educational plans (Additional file 1).

### The survey of the residents who graduated from Tokai University School of Medicine

The questionnaire survey about learning Kampo medicine was conducted by mail at the end of the 2-year residency (Additional file 2). Out of 103 residents who passed the National Medical Licensing Examination among the graduates of our medical school who graduated in March 2011, 93 residents, who had completed the survey in their fourth-year of medical school and confirmed the contact information, were surveyed. Because we had previously conducted a similar questionnaire survey of the students' awareness of Kampo medicine after their fourth-year Kampo lectures, at the Tokai University School of Medicine in 2008 [14], we required a signature on this questionnaire of the present survey to compare any changes in their awareness of Kampo medicine with the former questionnaire survey. The reply was sent by mail or facsimile. The residents who answered both questionnaires were analyzed.

To prepare both of the questionnaires, we used the semantic differential method, which is often applied in psychological research, to assure their validity [15]. To increase the response rate, we asked the hospitals and residents, who did not return responses, a second time for their completed questionnaires. The appropriate responsible persons from all the clinical training hospitals and all residents gave written informed consent to participate in this study. These surveys were approved by the Institutional Review Board for Clinical Research of Tokai University and conformed to the principles of the Helsinki Declaration.

### Statistical analysis

The Mann-Whitney *U* test was used to examine the relationship between the numbers of residents in each training hospital, i.e., hospital sizes and the implementation, educational methods and problems with introduction of Kampo education during their 2-year residency. Comparing the participants’ awareness of Kampo medicine during the fourth year of medical school with that at the end of their 2-year residency, only respondents to both questionnaires were registered in the present study. The Wilcoxon signed-rank test was used to compare any changes in awareness of Kampo medicine. The Chi-square test was used to analyze the current status of Kampo formulae use. All *p* values less than 0.05 were considered statistically significant.

## Results

### The survey of clinical training hospitals

#### Study population

There were 816 (81%) effective responses from 1011 hospitals, where 13,892 (90%) of all the 15,353 residents in Japan worked.

#### Instructors’ opinions

Of the answers from 815 instructors from the hospitals that were analyzed, regarding the necessity for Kampo clinical skills, 205 instructors (25%) generally thought that it was “Very necessary” for physicians to learn clinical skills of Kampo medicine, 481 (59%) thought it was “Slightly necessary,” 125 (15%) thought it was “Hardly necessary,” and 4 (1%) thought it was “Not necessary at all.” Therefore, 84% of the instructors thought that clinical physicians ought to be required to have clinical skills to use Kampo medicine.

For the introduction of Kampo education into the clinical training, 127 (16%) of 813 instructors thought it was “Very necessary,” 420 (51%) thought it was “Slightly necessary,” 253 (31%) thought it was “Hardly necessary,” and 13 (2%) thought it was “Not necessary at all.” Therefore, 67% of all the instructors had positive opinions regarding the introduction of Kampo education. In addition, 131 instructors (16%) thought that a standardized form of education was “Very necessary” for Kampo education during clinical training, 368 (45%) thought it was “Slightly necessary,” 300 (37%) thought it was “Hardly necessary,” and 14 (2%) thought it was “Not necessary at all,” revealing that 61% of the instructors thought there was a need for a standardized educational curriculum for Kampo medicine (Table 1).

**Table 1:** Instructors' opinions of Kampo medicine for residents

Variables	Respondents	Very	Slightly	Hardly	Not at all
Necessity for Kampo clinical skills	815	205 (25%)	481 (59%)	125 (15%)	4 (1%)
Necessity of introducing Kampo education into the clinical training	813	127 (16%)	420 (51%)	253 (31%)	13 (2%)
Necessity of a standardized educational curriculum for Kampo medicine	813	131 (16%)	368 (45%)	300 (37%)	14 (2%)

#### Implementation of Kampo education

**Fig. 1:**
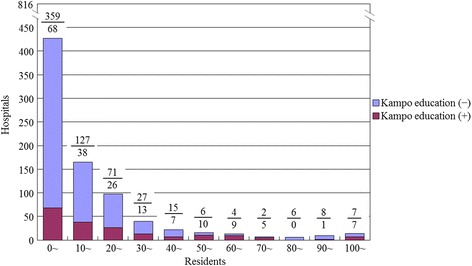
Numbers of training hospitals by hospital size (number of residents accepted at each hospital) and implementation of Kampo education. A/B:Number of training hospitals without (**a**) or with (**b**) Kampo education

Only 184 (23%) of the 816 responding hospitals teach Kampo medicine for residents, which translates to 4643 (33%) of 13,892 residents being able to receive Kampo education. The number of residents (first- and second-year) that each training hospital accepted was from 0 to 230 (median 8.5). If the hospitals with less than 10 residents were classified as small hospitals, 10 to 49 as medium hospitals, and 50 or more as large hospitals, 427 (52%) of 816 hospitals that were surveyed were small hospitals, 324 (40%) were medium hospitals, and 65 (8%) were large hospitals. Only 68 small hospitals (16%), 84 medium hospitals (26%) and 32 large hospitals (49%) taught Kampo medicine (Table 2). The larger hospitals that accepted more residents significantly had a high ratio of introducing Kampo medicine into their training programs than did the smaller hospitals (*p* < 0.001) (Fig. 1). The participation rate of residents to the Kampo study sessions was 0–20% in 46 hospitals (25%), 20–40% in 36 hospitals (20%), 40–60% in 37 hospitals (20%), 60–80% in 28 hospitals (15%), and 80–100% in 37 hospitals (20%).

**Table 2:** Kinds of current and future Kampo educatinal methods by hospital sizes

Variables			Respondents	Small hospitals	Medium hospitals	Large hospitals
All hospitals surveyed			816	427/816 (52%)	324/816 (40%)	65/816 (8%)
Kampo education (+)	Hospitals with Kampo education		184/816 (23%)	68/427 (16%)	84/324 (26%)	32/65 (49%)
	Current Kampo educational methods (multiple responses allowed)	Hospital independently	69/184 (38%)	22/68 (32%)	29/84 (35%)	18/32 (56%)
		Interhospital cooperation	15/184 (8%)	7/68 (10%)	5/84 (6%)	3/32 (9%)
		Kampo manufacturers	111/184 (60%)	42/68 (62%)	54/84 (64%)	15/32 (47%)
		Voluntary study	28/184 (15%)	15/68 (22%)	11/84 (13%)	2/32 (6%)
Kampo education (−)	Hospitals without Kampo education		632/816 (77%)	359/427 (84%)	240/324 (74%)	33/65 (51%)
	Future plans to introduce Kampo education (+)		72/622 (12%)	43/354 (12%)	23/236 (10%)	6/32 (19%)
	Future Kampo educational methods (multiple responses allowed)	Hospital independently	61/632 (10%)	25/359 (7%)	21/240 (9%)	15/33 (45%)
		Interhospital cooperation	201/632 (32%)	117/359 (33%)	76/240 (32%)	8/33 (24%)
		Kampo manufacturers	222/632 (35%)	142/359 (40%)	74/240 (31%)	6/33 (18%)
		Voluntary study	265/632 (42%)	154/359 (43%)	101/240 (42%)	10/33 (30%)

Hospital independently means, “Study sessions given by the hospital independentl”; Interhospital cooperation, “Study sessions given in cooperation with other hospitals”; Kampo manufacturers, “Lectures sponsored by Kampo manufacturers”; Voluntary study, “Voluntary study sessions given by physicians”

#### Kampo educational methods

Among 184 training hospitals that taught Kampo medicine, 69 hospitals (38%) answered that their Kampo education was organized by each hospital independently, by working in cooperation with other hospitals in 15 hospitals (8%), by Kampo manufacturers in 111 (60%), and by volunteer physicians in 28 hospitals (15%). Miscellaneous answers included by teaching in each clinical department in 4 hospitals and by practice in the Kampo clinic in 3 hospitals. Considering Kampo educational methods according to hospital size, Kampo education was organized by each hospital independently in 22 (32%) of 68 small hospitals, 29 (35%) of 84 medium hospitals, and 18 (56%) of 32 large hospitals; by working in cooperation with other hospitals in 7 (10%), 5 (6%), and 3 (9%); by Kampo manufacturers in 42 (62%), 54 (64%), and 15 (47%); and by volunteer physicians in 15 (22%), 11 (13%), and 2 (6%), respectively (Table 2). The hospitals teaching Kampo medicine with such proactive methods as study sessions organized by each hospital independently and study sessions given in cooperation with other hospitals accepted significantly more residents than did those that had passive forms of Kampo education, such as lectures sponsored by Kampo manufacturers and voluntary study sessions given by physicians (*p* = 0.032).

#### Reasons not to teach Kampo medicine

**Fig. 2:**
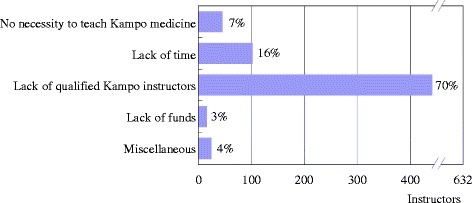
Reasons not to teach Kampo medicine

Regarding the reasons not to teach Kampo medicine, among 632 instructors at the hospitals without Kampo education, “the lack of qualified Kampo instructors” was pointed out most by 442 instructors (70%), “the lack of time” by 103 (16%), “no necessity to teach Kampo medicine” by 46 (7%), and “the lack of funds” by 16 (3%) (Fig. 2). Miscellaneous answers included “the high priority to teach Western medicine” by 15 instructors, “no standardized educational curriculum” by 5, and “the lack of clinical evidence” by 2. The 442 instructors who pointed out “the lack of qualified Kampo instructors” included 264 (74%) of 359 instructors of small hospitals, 159 (66%) of 240 instructors of medium hospitals and 19 (58%) of 33 instructors of large hospitals. “The lack of qualified Kampo instructors” was a more serious problem in smaller hospitals than it was in larger hospitals (*p* = 0.011).

#### Future Kampo education

There was a total of 622 of 632 hospitals without Kampo education programs that answered. Only 72 (12%) of them planned to teach Kampo medicine in their clinical training programs, and the breakdown of those was 43 (12%) of 354 small hospitals, 23 (10%) of 236 medium hospitals, and 6 (19%) of 32 large hospitals. Overall, 550 (67%) of 816 hospitals that responded had no Kampo education programs and no plans to introduce any such programs in the future, and the rate of the hospitals without Kampo education programs currently and in the future was higher in small hospitals (*p* < 0.001).

Regarding the Kampo educational methods they wanted to develop in their hospitals in the future, “Study sessions given by the hospital independently” was only selected by 61 hospitals (10%) among 632 hospitals without Kampo education, “Study sessions given in cooperation with other hospitals” was selected by 201 hospitals (32%), “Lectures sponsored by Kampo manufacturers” by 222 hospitals (35%), and “Voluntary study sessions given by physicians” was selected most by 265 hospitals (42%). Miscellaneous answers included: “Looking for qualified instructors” by 8 hospitals, “Lectures given by medical associations,” “Introduction to the training program” and “Training for the senior residents” by 7, each, “Individual guidance in the routine medical training” by 4, and “E-learning” by 2. Considering these results with each hospital size, 25 (7%) of 359 small hospitals, 21 (9%) of 240 medium hospitals, and 15 (45%) of 33 large hospitals would hold study sessions by the hospital independently. Furthermore, study sessions given in cooperation with other hospitals were selected by 117 (33%), 76 (32%), and 8 hospitals (24%); lectures sponsored by Kampo manufacturers by 142 (40%), 74 (31%) and 6 (18%); voluntary study sessions given by physicians by 154 (43%), 101 (42%), and 10 (30%) among small, medium, and large hospitals, respectively (Table 2). These results revealed that larger hospitals were more proactive in teaching Kampo medicine compared with smaller hospitals (*p* = 0.014), and “study sessions by the hospital independently” are held more in larger hospitals, and “study sessions in cooperation with other hospitals” are held more in smaller hospitals (*p* < 0.001).

### Survey of the residents

#### Study population

There were 72 (37 males and 35 females) effective responses from 93 residents. The response rate was 77%.

#### Attitudes toward Kampo medicine

**Fig. 3:**
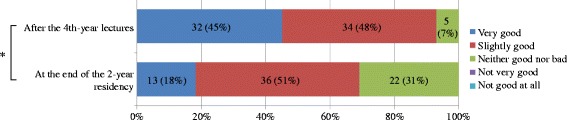
Comparison of general impression of Kampo medicine after the fourth-year lectures and at the end of the 2-year residency. Wilcoxon signed-rank test, *n* = 71, *p < 0.001

**Fig. 4:**
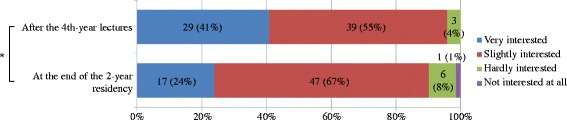
Comparison of interest in Kampo medicine after the fourth-year lectures and at the end of the 2-year residency. Wilcoxon signed-rank test, *n* = 71, *p = 0.002

The number of residents who answered that their general impression of Kampo medicine was good at the end of their 2-year residency was 49 (69%). Overall, their impression worsened significantly compared with that when they were fourth-year medical students (*p* < 0.001) (Fig. 3), and 64 residents (91%) who were interested in Kampo medicine also evidenced that they significantly lost interest (*p* = 0.002) (Fig. 4). However, the residents who had opportunities to learn Kampo medicine during their residency showed no changes in their impression of it and interest in it before and after their residency; but the impression of Kampo medicine of those who only had limited opportunities to learn it significantly worsened (*p* < 0.001), and their interest in Kampo medicine declined (*p* = 0.016). Regarding the value of learning Kampo medicine, almost all residents (96%) recognized that Kampo medicine was worth learning, including: “Very much” by 32 (45%) and “Slightly” by 36 (51%). No one thought “Hardly” or “Not at all,” and only 3 (4%) answered “No idea.”

#### Methods and impressions of the Kampo training

**Fig. 5:**
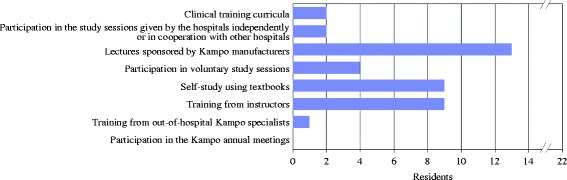
Methods of learning Kampo medicine (multiple responses allowed)

There were 22 (31%) of 70 residents who had opportunities to learn Kampo medicine during their residency. The study methods were mainly voluntary learning such as taking lectures sponsored by Kampo manufacturers. Only 4 residents received Kampo education that their training hospital regularly offered: 2 residents received clinical training in Kampo medicine, and 2 other residents had chances to participate in the study sessions given by the hospital independently or in coordination with the cooperation of other hospitals (Fig. 5). Regarding the satisfaction of the Kampo training during the 2-year residency: 4 (19%) of 21 residents were very satisfied, 6 (29%) were slightly satisfied, 9 (42%) were hardly satisfied, and 2 (10%) were not satisfied at all. In addition, 8 (17%) of 47 residents who only had limited opportunities to study Kampo medicine wanted to study it very much, 31 (66%) slightly wanted to study it, 7 (15%) hardly wanted to study it, and 1 (2%) did not want to study it at all. That is, a total of 39 (83%) residents wanted to study Kampo medicine during residency.

Fourth-year students at Tokai University School of Medicine have 6 h of Kampo lectures and 3 h of Kampo experience-based learning [14]. To the question of whether or not these Kampo lectures were helpful in daily clinical practice, 6 (9%) of 70 residents answered “Very helpful,” 29 (41%) “Slightly helpful,” 29 (41%) “Hardly helpful,” and 6 (9%) “Not helpful at all.” Because 50% of all the residents thought the lectures helpful, undergraduate Kampo education was considered to be good for developing competent physicians.

#### Grounds for prescribing Kampo medicine

Among 71 residents who were surveyed, almost all (66, 93%) had prescribed Kampo medicine during their 2-year residency, and only 5 (7%) had never prescribed it. Out of 65 residents who had prescribed Kampo medicine, 47 (72%) had done so because of suggestions from instructors, 20 (31%) had experiences of prescribing Kampo medicine at their own discretion. A miscellaneous answer was that the physician followed the advice of a pharmacist.

**Fig. 6:**
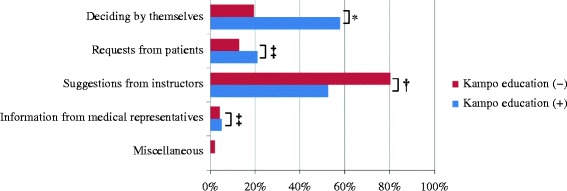
Comparison of grounds for prescribing Kampo medicine between residents without Kampo education and those with Kampo education. Chi-square test, *p = 0.003, †*p* = 0.037, ‡ = n.s

Residents who were taught Kampo medicine during their residency prescribed Kampo medicine more actively (*p* = 0.003) using their own discretion than did those without Kampo education. Those who did not have Kampo education, however, had a relatively passive attitude against prescribing Kampo medicine and, in many cases, prescribed it according to their instructors’ suggestions (*p* = 0.037) (Fig. 6).

#### Future expectations of Kampo medicine

Approximately 73% of the residents thought that Kampo education should be introduced into the curriculum. The breakdown revealed: 13 (19%) of 70 residents thought “Very much,” 38 (54%) thought “A little,” 18 (26%) thought “Not so much,” and 1 (1%) thought “Not at all.”

**Fig. 7:**
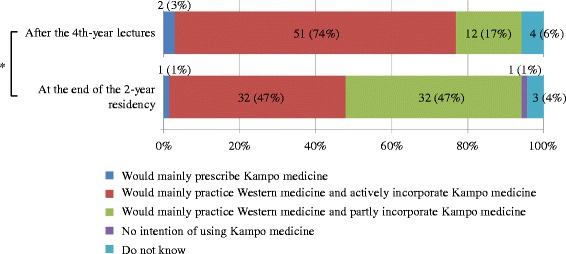
Changes in awareness of prescribing Kampo medicine after the fourth-year lectures and at the end of the 2-year residency. Wilcoxon signed-rank test, *n* = 69, *p < 0.001

We examined the changes in the awareness of prescribing Kampo medicine between after the students’ fourth-year Kampo lectures and at the end of their 2-year residency. Among 69 residents who responded to both questionnaires, 7 answered that they had “no idea” of prescribing Kampo medicine in the future in either or both questionnaire(s), and the remaining 62 were examined for the changes. The results revealed that the physicians statistically became reluctant to prescribe Kampo medicine after their residency (*p* < 0.001) (Fig. 7).

To the last question, of whether or not it was necessary to practice the Japanese-style integrated medicine that incorporates the traditional Japanese medicine, of acupuncture, moxibustion, and Kampo medicine, into the conventional (Western-style) medicine in the future, 19 (27%) residents considered it “Very necessary,” and 43 (62%) thought it was “Slightly necessary.” There were very few negative opinions. Only 8 (11%) residents thought it was “Hardly necessary,” and no one thought it was “Not necessary at all.”

## Discussion

Recently, a few survey reports were published on the status of undergraduate Kampo education offered at some Japanese medical schools [10, 16, 17]. The present study revealed that most medical students were only offered a short time to study Kampo medicine mainly because of the lack of qualified instructors to teach Kampo medicine and the non-standard curricula [10]. Regarding medical students’ awareness of Kampo medicine, we reported that almost all medical students realized the necessity of learning Kampo medicine and really wanted to learn it in their 2-year clinical training [18]. Compared to students of other medical related professions, however, medical students were reported to be passive regarding CAM [19], likely because they received scant proper CAM education. Therefore, CAM including Kampo medicine should be taught not only in medical schools but also during residency.

Nowadays, many Japanese doctors use Kampo medicine in clinical practice [6] and prescribe Kampo extract medicine together with the treatment of Western medicine in many cases. In modern medicine, of which the mainstream is Western medicine, Kampo medicine should be practically integrated into the conventional Western medicine like other CAM education in other areas [20–22]. Actually in the education during residency, it is not only necessary to introduce Kampo medicine into their clinical training programs but also to teach clinical evidence of Kampo medicine that supports the efficacy of Kampo treatments [23]. This new type of medical treatment that integrates Kampo medicine and conventional Western medicine may be referred to as a kind of Japanese-style integrative medicine. For the establishment of this type of medicine, we first have to investigate the current status of Kampo education for residents and determine what the problems are in order to arrive at effective solutions for the future.

The present study is composed of two surveys about Kampo education: one is a nationwide survey of all 1011 training hospitals in Japan where the Kampo training is conducted, and the other is a questionnaire survey on the changes in awareness of Kampo medicine of the students who graduated from Tokai University School of Medicine in 2011 between after their fourth-year Kampo lectures and after their 2-year residency. There are scant reports on the present status of Kampo education in clinical training in Japan, other than our own pilot study of 58 training hospitals in Kanagawa prefecture adjacent to and southwest of Tokyo [13]. Regarding residents’ awareness of Kampo medicine, very little is known because, preceding the present study, there have been no reports on this issue.

The results of the survey of the training hospitals must be quite reliable because it was a nationwide investigation with the high effective response rate of 81%, representing 90% of all the residents surveyed, and all the other participating respondents who were the chief instructors responsible for the medical education in each hospital. Regarding the survey of the residents, the 31% rate of residents who learned Kampo medicine was near the 33% of residents who had been taught it according to the results of our survey of all the hospitals. These two questionnaires also represented two aspects of the implementation rate of Kampo education during the 2-year residency. Because the implementation rates from the two viewpoints were almost the same, the group of residents who were analyzed might have been a good representative sample of all Japanese residents, even though it was a pilot study limited to the graduates from Tokai University School of Medicine 5 years ago in 2011. Therefore, the findings obtained from the present survey were also considered to be reliable.

Our surveys are, therefore, unique and significant in three aspects. First, these surveys are the first extensive investigations of Kampo education during residency in Japan, even though one of the surveys was a pilot study limited to the residents who graduated from Tokai University School of Medicine in 2011. Next, they reveal, for the first time, the current status and problems related to Kampo education during residency from the two main, critical viewpoints of training hospitals and residents. And finally, this survey of the residents is the first study that compares the changes, before and after their residency, in their awareness of Kampo medicine.

We initially clarified the current issues of the Kampo educational system in the training hospitals throughout Japan. Our survey revealed that 84% of instructors thought it necessary for physicians to learn the requisite clinical skills to prescribe Kampo medicine, 67% thought it necessary to introduce Kampo education into the clinical training, and 61% thought that a standardized educational curriculum for Kampo medicine was needed. Most instructors recognize that Kampo therapies are needed in clinical practice and that Kampo medicine should be taught systematically during residency. Furthermore, 91% of the residents were interested in Kampo medicine, 73% thought that Kampo medicine should be introduced into the training programs, and 50% would actively incorporate Kampo medicine into Western medicine, in their own practices, in the future. However, only 23% of the training hospitals currently teach Kampo medicine, which includes, at most, 33% of the residents. The reason that Kampo education is not widely offered might likely be due to the same situation as that of CAM education in other areas [24]. Regarding the reasons for the infrequent opportunities to learn Kampo medicine in clinical training, it was pointed out that Kampo medicine is not described in the educational goals of any clinical training program and that most instructors have never received formal training in Kampo medicine [25]. This opinion supports our suggestion that it is important to develop a basic environment in training hospitals to teach Kampo medicine, such as training qualified instructors, introducing Kampo medicine into training programs, and setting Kampo educational goals to spread Kampo medicine in clinical training.

We subsequently examined the timing for introducing Kampo education during residency. Most residents who were surveyed in this study had only received Western medicine training and only had limited opportunities to learn Kampo medicine for approximately 4.5 years since the fourth-year Kampo lectures. Perhaps the lack of Kampo education mainly caused overall deterioration of their general impression and the decline in interest in Kampo medicine. However, residents who learned Kampo medicine during their residency maintained good impressions and interest in it and actively prescribed it at their own discretion; but those who did not learn it, significantly had weaker impressions of it, exhibited less interest in it, and only prescribed it reluctantly according to requests and/or suggestions from others. The reasons for this may not only be that residents who have not had Kampo education have little knowledge of it and lack the requisite skills to use it effectively, and have only minimal motivation to learn it, but also that the instructors at the training hospitals that do not provide Kampo education lack the understanding of Kampo medicine, have negative attitudes toward Kampo education, and do not want residents to prescribe Kampo medicine. In addition, the 2-year residency is not only their first training period as doctors but also an important time to help form clinicians’ attitudes [26]. Therefore, teaching Kampo medicine to highly motivated residents who want to learn it during their residency is recommended to help them develop even broader clinical knowledge and skills.

The hospital sizes that would be adequate to teach Kampo medicine were also reported. Seventy percent of the hospitals that did not have Kampo education as part of their clinical training indicated that the main reason they did not was because there were too few qualified instructors who could teach it, and that fact was even more serious in smaller hospitals. In Kampo education for undergraduates, the fact that there are relatively few medical schools that hire full-time, qualified, instructors to teach Kampo medicine has seemed to greatly impede the general improvement of Kampo education [4]. Our prior survey also revealed that problem, for the majority of medical schools, that of finding and training instructors who would be qualified and responsible for teaching Kampo medicine [10]. Our survey revealed that Kampo education was introduced more widely in larger hospitals. That is likely because there are so many physicians in large hospitals that it may be easier to find well-qualified Kampo instructors, who have adequate qualities and accomplishments to be able to teach Kampo medicine. Therefore, to effectively include Kampo medicine into residents’ education, it will first be important to introduce it into the training curricula of large hospitals [13].

Adequate methods to introduce Kampo education to residents of small hospitals must likewise be discussed. To that end, it will be necessary to examine reasonable educational forms of Kampo medicine that correspond to each hospital’s size because Kampo education is currently conducted in many different forms [27]. Most of the large hospitals with Kampo education have positive educational forms such as study sessions given by the hospital independently (56%) and those without it had plans to do so in the future (45%). However, in 84% of the small hospitals with Kampo education, they tended to have passive forms of Kampo education, such as lectures sponsored by Kampo manufacturers and voluntary study sessions given by physicians, and 83% of those currently without it only had plans for passive forms in the future. The present survey additionally revealed that only 12% of the hospitals planned to introduce Kampo education in the near future, and 67% of the training hospitals surveyed had no current Kampo education nor did they have any future plans for it. This tendency was significant in the smaller hospitals. These passive attitudes toward the introduction of Kampo education might mainly be caused by the difficulty of being unable to find qualified instructors in small hospitals even though Kampo education could be introduced into their training programs. Therefore, it will be challenging for the small hospitals, of which there is a majority, and most of which currently have no Kampo education at all, to introduce Kampo education into their curricula, which would be provided by the hospitals themselves.

To solve this problem of the limited availability of qualified Kampo instructors, as a viable model, relatively small hospitals could cooperate with other hospitals for the purposes of providing their staff with Kampo education [28]. Actually, this method of education is conducted in only 15 hospitals in Japan, but it will soon be introduced in 201 other hospitals, which represent 32% of the training hospitals without Kampo education, and will likely become a feasible model of Kampo education during residency in the near future. If the interhospital Kampo education system is difficult to organize, for example, establishment of web-based training courses [29, 30] and cooperation with hospitals that can receive Kampo trainees at affiliated hospitals [31] may be possible solutions.

The participation rate of residents to the Kampo study sessions was considerably different at each training hospital. A low participation rate of Kampo study sessions suggested that the concepts of Kampo training could be less interesting for residents. Previous reports showed that introducing experience-based learning is effective to teach CAM that includes Kampo medicine [14, 32], and most residents were interested in Kampo medicine, Kampo case studies, and the Kampo-style of the abdominal examination [33]. Recently an abdominal palpation educational simulator was developed and expected to be introduced into Kampo education [34, 35]. In addition, Kampo medicine is clinically unique compared to other kinds of CAM in terms of its being prescribed by physicians practicing Western medicine, and sometimes in combination with Western-style medicine in Japan [5, 36].

Considering these aspects, more appealing Kampo training programs should be developed to improve the residents’ motivation, who will mainly practice Western medicine, toward learning to use Kampo medicine in their combined therapies. Therefore, to spread Kampo education for residents nationwide, establishing standard programs and guidelines for it are issues that must be resolved at the same time as finding and fostering qualified Kampo instructors.

There are some limitations of this study. First, the data collection took a long time because we repeatedly asked some training hospitals and residents who had not returned their completed questionnaires to please complete them and return them. Because the questionnaire for the training hospitals asked questions regarding the status of Kampo education in 2013 and that for the residents at the end of their 2-year residency, this delay might have affected the answers of some hospitals and residents that replied to the questionnaire and/or returned it at a later date. Another limitation was that the residents surveyed were limited to the graduates from the Tokai University School of Medicine in 2011. However small or biased this population might have been, we accurately compared the graduates’ awareness of Kampo medicine after their fourth-year lectures with that at the end of their 2-year residency. And the final limitation could be that the actual curricula contents of Kampo education were not examined in this study because the aim of the study was to survey the current status of Kampo education during residency, not the quality and quantity of it, to identify the major problems, and to suggest solutions in order to better promote Kampo education during the 2-year residency and beyond. Further studies are, therefore, warranted after re-examining these issues to establish more adequate procedures and curricula for Kampo education.

## Conclusions

Our study revealed that most instructors at training hospitals in Japan understood the necessity of Kampo education, however, little of it was actually done mainly because of the lack of Kampo instructors, especially in small hospitals. Our results also revealed that most residents recognized the need for Kampo medicine and are highly motivated to learn it; however, they actually, mostly prescribed Kampo medicine in response to suggestions from their instructors. This is likely due to the limited opportunity for residents to learn Kampo medicine; therefore, we conclude that Kampo education should be introduced during residency. And for small hospitals where there is a lack of Kampo instructors, cooperating with other hospitals, as a method of teaching Kampo medicine, was considered a promising effective solution.

## Additional files

Additional file 1: Questionnaire on the current Kampo education in clinical training. (DOCX 21 kb)
Additional file 2: Questionnaire after residency about learning Kampo medicine. (DOCX 29 kb)

## Abbreviation

CAM: Complementary and alternative medicine
