# Supplementary material for: The education of traditional Japanese (Kampo) medicine: surveys of training hospitals and residents
Source: BMC Complement Altern Med. 2017 Mar 2;17:134. doi: 10.1186/s12906-017-1634-2 (PMC5335720; doi:10.1186/s12906-017-1634-2)
Supplement: Additional file 1: — Questionnaire on the current Kampo education in clinical training. (DOCX 21 kb) [file 12906_2017_1634_MOESM1_ESM.docx]

Questionnaire on the current Kampo education in clinical training

1. How many clinical residents are there in your hospital?

N = ______ first-year physicians

N = ______ second-year physicians

1. Is it necessary for physicians to learn clinical skills regarding Kampo medicine?
   1. Very necessary
   2. Slightly necessary
   3. Hardly necessary
   4. Not necessary at all
2. Is it necessary to introduce Kampo education into the clinical training?
3. Very necessary
4. Slightly necessary
5. Hardly necessary
6. Not necessary at all
7. Are standardized educational curricula necessary for Kampo education in clinical training?
8. Very necessary
9. Slightly necessary
10. Hardly necessary
11. Not necessary at all
12. Do you teach Kampo medicine during clinical training?
13. YES　　　　 → Go on to Question 6.
14. NO　　　　 → Go on to Question 8.

If YES in Question 5), please answer the following questions.

1. Who organized the Kampo education? (multiple responses allowed)
2. One hospital working independently
3. Hospitals working in cooperation with other hospitals
4. Kampo manufacturers
5. Volunteer physicians
6. Other (Specify: )
7. What is the rate of participation of residents to the Kampo study sessions?
8. 0−20%
9. 20−40%
10. 40−60%
11. 60−80%
12. 80−100%

If NO in Question 5, please answer the following questions.

1. Why have you not introduced Kampo education? Please select one main reason.
2. No necessity to teach Kampo medicine
3. Lack of time
4. Lack of qualified Kampo instructors
5. Lack of budget
6. Other (Specify: )
7. Do you intend to introduce Kampo education in the future?
8. YES
9. NO
10. How do you want to develop Kampo education in your hospital? (multiple responses allowed)
11. Study sessions given by the hospital independently
12. Study sessions given in cooperation with other hospitals
13. Lectures sponsored by Kampo manufacturers
14. Voluntary study sessions given by physicians
15. Other (Specify: )

This is the end of the questions.

Name:

Affiliation:
