# Supplementary material for: The education of traditional Japanese (Kampo) medicine: surveys of training hospitals and residents
Source: BMC Complement Altern Med. 2017 Mar 2;17:134. doi: 10.1186/s12906-017-1634-2 (PMC5335720; doi:10.1186/s12906-017-1634-2)
Supplement: Additional file 2: — Questionnaire after residency about learning Kampo medicine. (DOCX 29 kb) [file 12906_2017_1634_MOESM2_ESM.docx]

Questionnaire after residency about learning Kampo medicine

1. What is your general impression of Kampo medicine?
   1. Very good
   2. Slightly good
   3. Neither good nor bad
   4. Not very good
   5. Not good at all
2. Are you interested in Kampo medicine?
   1. Very interested
   2. Slightly interested
   3. Hardly interested
   4. Not interested at all
3. Is Kampo medicine worth learning?
   1. Very much worth learning
   2. Slightly worth learning
   3. Hardly worth learning
   4. Not worth learning at all
   5. No idea
4. Were the lectures on Kampo medicine when you were a medical student helpful to you now in daily clinical practice?
5. Very helpful
6. Slightly helpful
7. Hardly helpful
8. Not helpful at all
9. Did you have an opportunity to learn Kampo medicine during your residency?
10. YES　　　　 → Go on to Question 6.
11. NO　　　　 → Go on to Question 8.

If YES in Question 5, please answer the next questions.

1. How did you learn about Kampo medicine? (Multiple responses allowed)
2. Clinical training curriculum
3. Participation in study sessions held by the hospital independently or in cooperation with other hospitals
4. Lectures sponsored by Kampo manufacturers
5. Participation in voluntary study sessions
6. Self-study using textbooks
7. Guidance from instructors
8. Guidance from out-of-hospital Kampo specialists
9. Participation in annual meetings on Kampo medicine
10. Other (Specify: )
11. Were you satisfied with the training on Kampo medicine in your residency?
12. Very satisfied
13. Slightly satisfied
14. Hardly satisfied
15. Not satisfied at all

→ Go on to Question 9.)

If NO in Question 5), please answer the next questions.

1. Did you want to have an opportunity to study Kampo medicine?
2. Very much
3. Slightly
4. Hardly
5. Not at all

→ Go on to Question 9.)

1. Have you prescribed Kampo medicine during your internship?
2. YES　　　　 → Go on to Question 10.)
3. NO　　　　 → Go on to Question 11.)

If YES in Question 9, please answer the next questions.

1. What was the main reason you prescribed Kampo medicine? (multiple responses allowed)
2. Decision by yourself
3. Request from the patient
4. Request from instructors
5. Information from medical representatives
6. Other (Specify: )

→ Go on to Question 12.

If NO in Question 9, please answer the next questions.

1. Why did you not prescribe Kampo medicine?
2. Not interested in Kampo medicine
3. Should prioritize the training regarding Western medicine
4. Not confident in the selection of the prescription
5. No available Kampo medicine to be prescribed
6. Not allowed by instructors
7. Not allowed by the hospital
8. Other (Specify: )

→ Go on to Question 12.

1. Should Kampo medicine be introduced into a training curriculum?
2. Very much
3. A little
4. Not so much
5. Not at all
6. Will you prescribe Kampo medicine in the future?
7. Will mainly prescribe Kampo medicine
8. Will mainly prescribe Western medicine and actively incorporate Kampo medicine
9. Will mainly prescribe Western medicine and partly incorporate Kampo medicine
10. No intention of prescribing Kampo medicine
11. No idea
12. Is it necessary to practice Japanese-style integrated medicine that incorporates the traditional Japanese medicine, such as acupuncture, moxibustion, and Kampo medicine, into the conventional (Western-style) medicine in the future?
13. Very necessary
14. Slightly necessary
15. Hardly necessary
16. Not necessary at all

This is the end of the questions.

Name:

Affiliation:

Course: Internal medicine ・ Surgical ・ Other ( )
